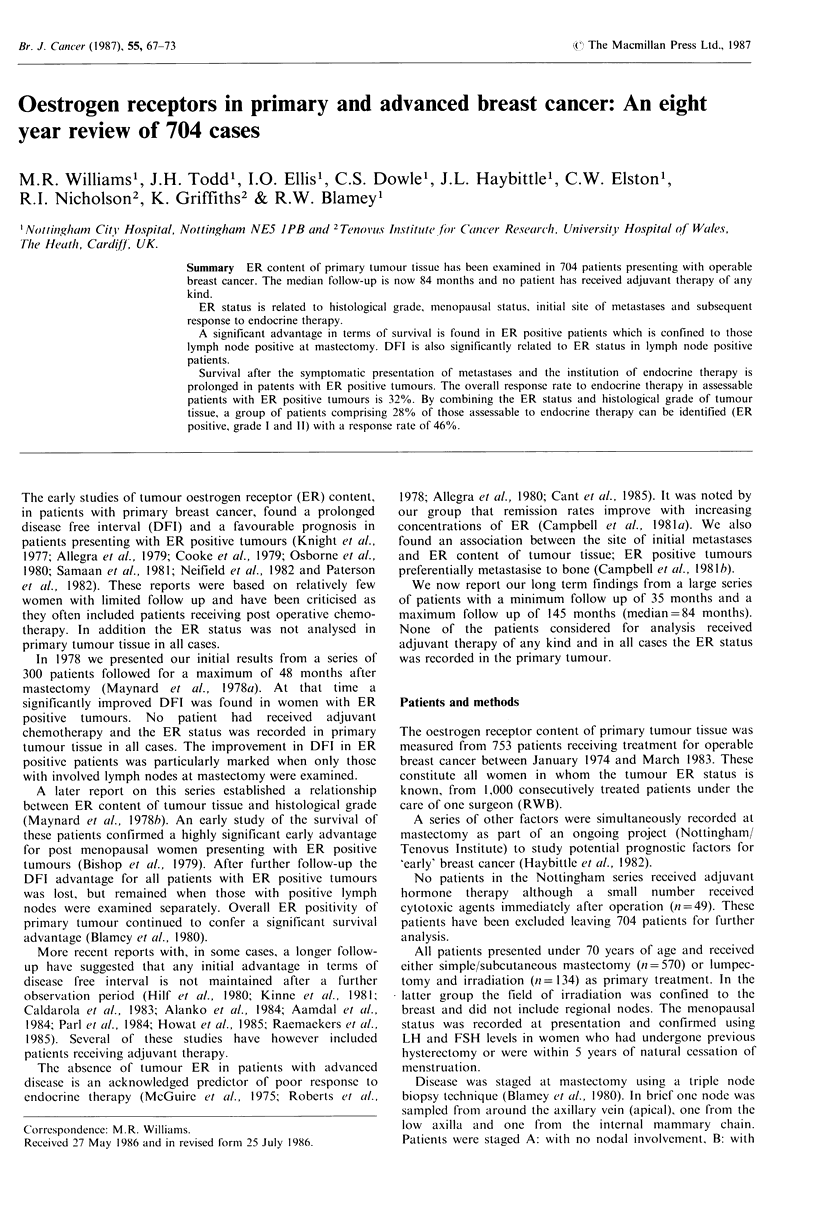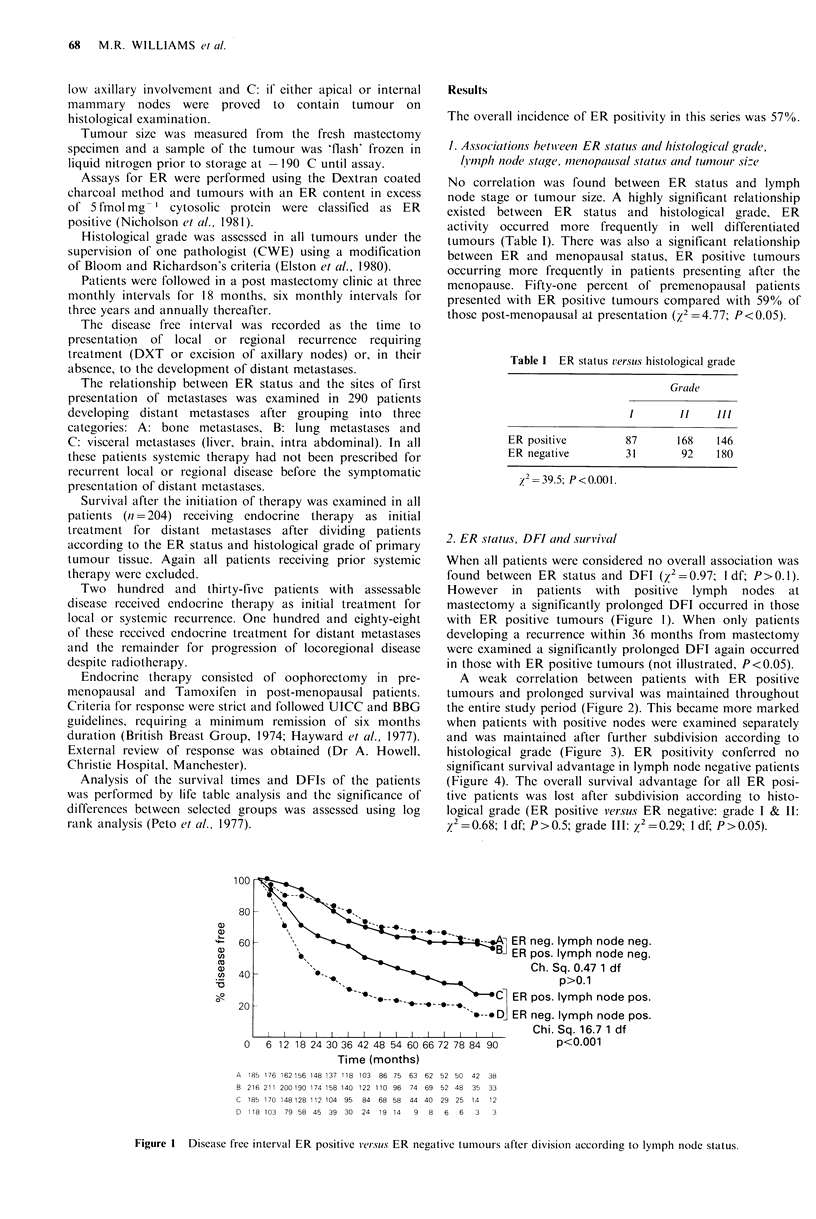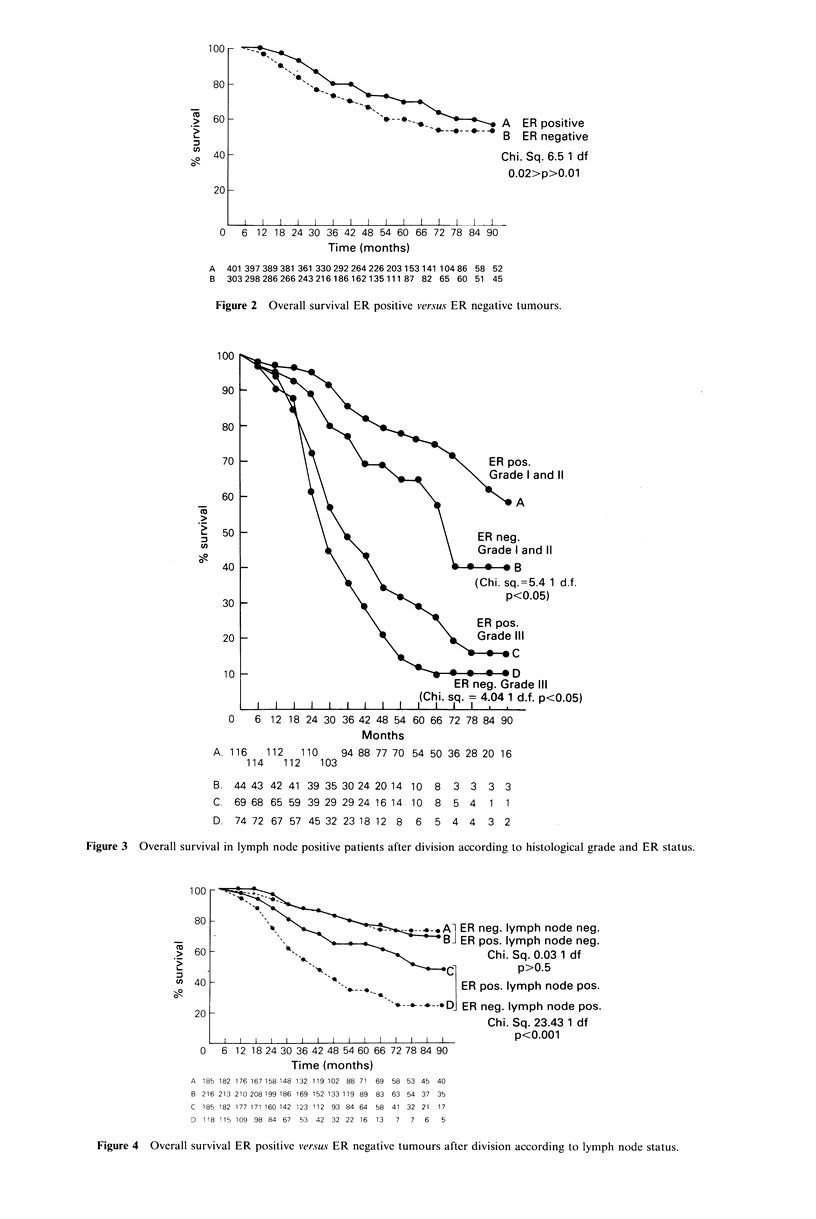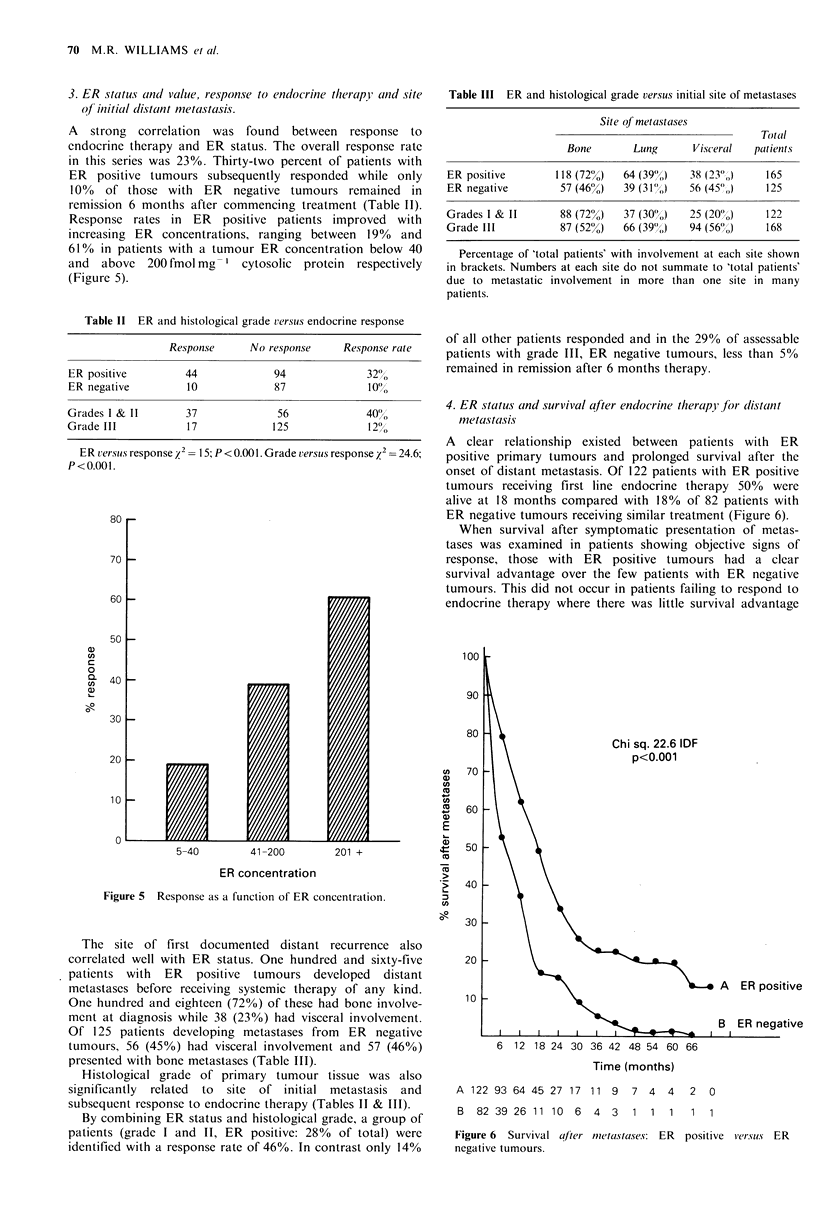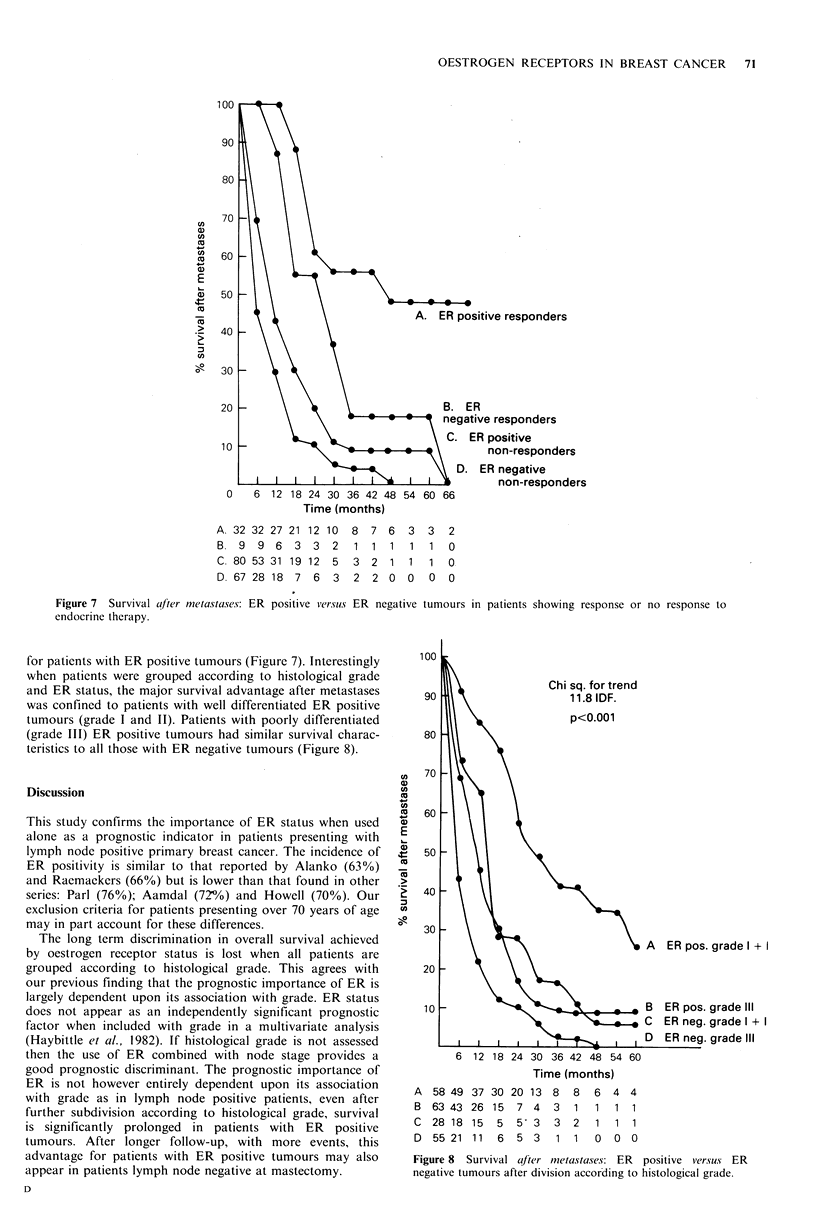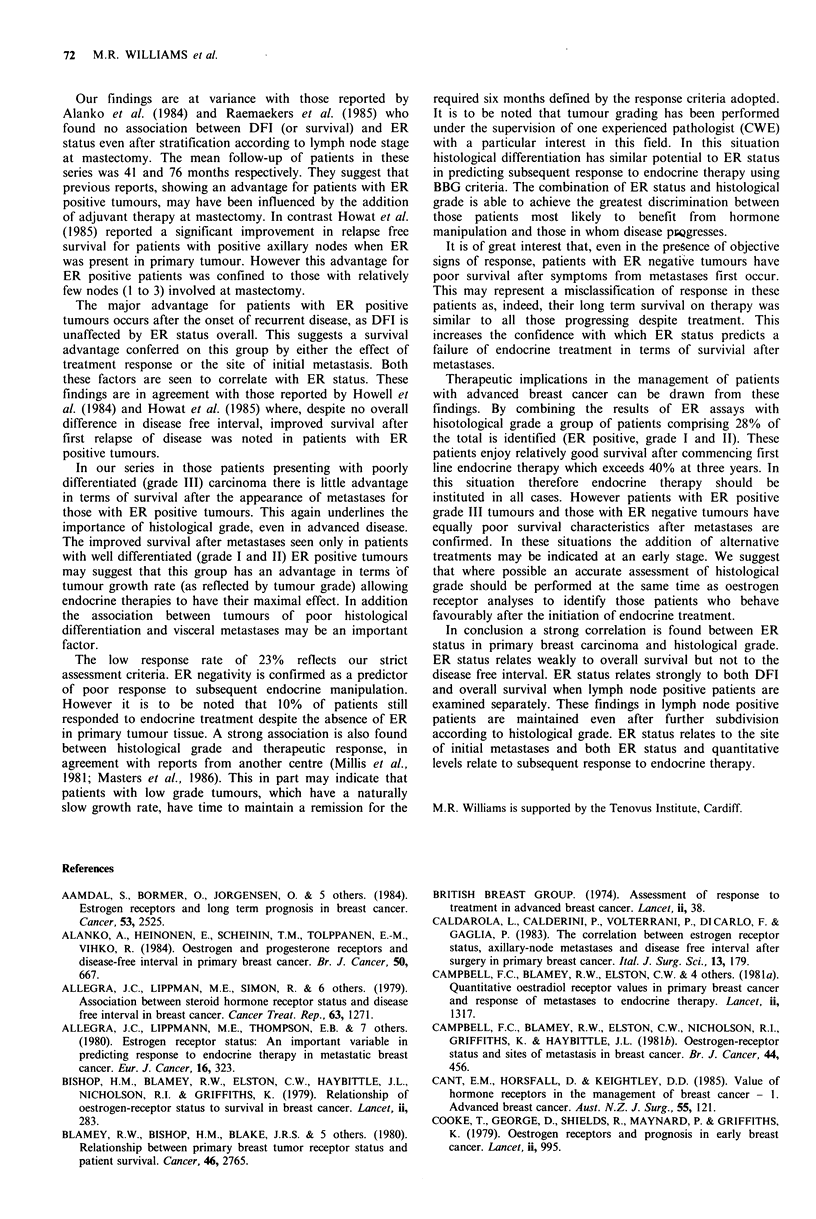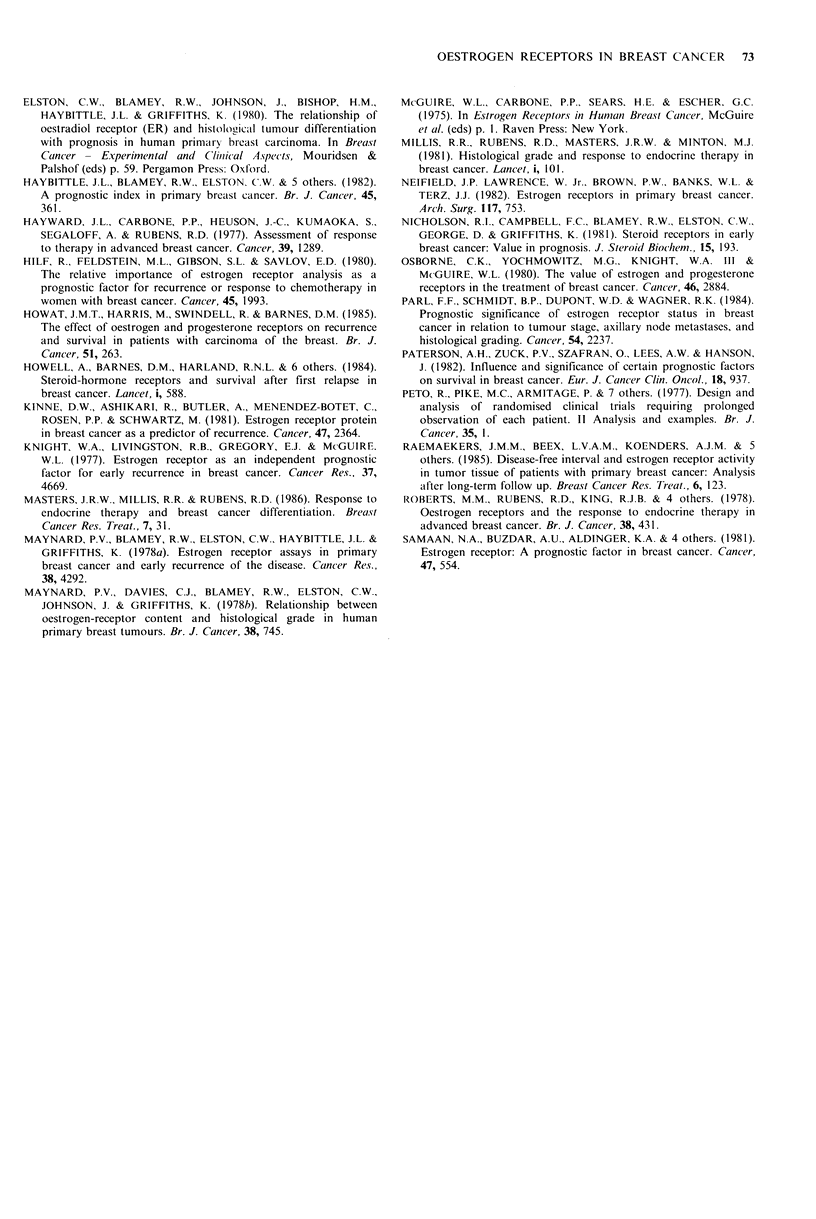# Oestrogen receptors in primary and advanced breast cancer: an eight year review of 704 cases.

**DOI:** 10.1038/bjc.1987.14

**Published:** 1987-01

**Authors:** M. R. Williams, J. H. Todd, I. O. Ellis, C. S. Dowle, J. L. Haybittle, C. W. Elston, R. I. Nicholson, K. Griffiths, R. W. Blamey

## Abstract

ER content of primary tumour tissue has been examined in 704 patients presenting with operable breast cancer. The median follow-up is now 84 months and no patient has received adjuvant therapy of any kind. ER status is related to histological grade, menopausal status, initial site of metastases and subsequent response to endocrine therapy. A significant advantage in terms of survival is found in ER positive patients which is confined to those lymph node positive at mastectomy. DFI is also significantly related to ER status in lymph node positive patients. Survival after the symptomatic presentation of metastases and the institution of endocrine therapy is prolonged in patients with ER positive tumours. The overall response rate to endocrine therapy in assessable patients with ER positive tumours is 32%. By combining the ER status and histological grade of tumour tissue, a group of patients comprising 28% of those assessable to endocrine therapy can be identified (ER positive, grade I and II) with a response rate of 46%.


*Br. J. Cancer* (1987), **55**, 67–73 © The Macmillan Press Ltd., 1987

### Oestrogen receptors in primary and advanced breast cancer: An eight year review of 704 cases

M.R. Williams[1], J.H. Todd[1], I.O. Ellis[1], C.S. Dowle[1], J.L. Haybittle[1], C.W. Elston[1], R.I. Nicholson[2], K. Griffiths[2] & R.W. Blamey[1]

[1]*Nottingham City Hospital, Nottingham NE5 1PB and* [2]*Tenovus Institute for Cancer Research, University Hospital of Wales, The Heath, Cardiff, UK.*

**Summary** ER content of primary tumour tissue has been examined in 704 patients presenting with operable breast cancer. The median follow-up is now 84 months and no patient has received adjuvant therapy of any kind.

ER status is related to histological grade, menopausal status, initial site of metastases and subsequent response to endocrine therapy.

A significant advantage in terms of survival is found in ER positive patients which is confined to those lymph node positive at mastectomy. DFI is also significantly related to ER status in lymph node positive patients.

Survival after the symptomatic presentation of metastases and the institution of endocrine therapy is prolonged in patents with ER positive tumours. The overall response rate to endocrine therapy in assessable patients with ER positive tumours is 32%. By combining the ER status and histological grade of tumour tissue, a group of patients comprising 28% of those assessable to endocrine therapy can be identified (ER positive, grade I and II) with a response rate of 46%.

The early studies of tumour oestrogen receptor (ER) content, in patients with primary breast cancer, found a prolonged disease free interval (DFI) and a favourable prognosis in patients presenting with ER positive tumours (Knight *et al.*, 1977; Allegra *et al.*, 1979; Cooke *et al.*, 1979; Osborne *et al.*, 1980; Samaan *et al.*, 1981; Neifield *et al.*, 1982 and Paterson *et al.*, 1982). These reports were based on relatively few women with limited follow up and have been criticised as they often included patients receiving post operative chemotherapy. In addition the ER status was not analysed in primary tumour tissue in all cases.

In 1978 we presented our initial results from a series of 300 patients followed for a maximum of 48 months after mastectomy (Maynard *et al.*, 1978*a*). At that time a significantly improved DFI was found in women with ER positive tumours. No patient had received adjuvant chemotherapy and the ER status was recorded in primary tumour tissue in all cases. The improvement in DFI in ER positive patients was particularly marked when only those with involved lymph nodes at mastectomy were examined.

A later report on this series established a relationship between ER content of tumour tissue and histological grade (Maynard *et al.*, 1978*b*). An early study of the survival of these patients confirmed a highly significant early advantage for post menopausal women presenting with ER positive tumours (Bishop *et al.*, 1979). After further follow-up the DFI advantage for all patients with ER positive tumours was lost, but remained when those with positive lymph nodes were examined separately. Overall ER positivity of primary tumour continued to confer a significant survival advantage (Blamey *et al.*, 1980).

More recent reports with, in some cases, a longer follow-up have suggested that any initial advantage in terms of disease free interval is not maintained after a further observation period (Hilf *et al.*, 1980; Kinne *et al.*, 1981; Caldarola *et al.*, 1983; Alanko *et al.*, 1984; Aamdal *et al.*, 1984; Parl *et al.*, 1984; Howat *et al.*, 1985; Raemaekers *et al.*, 1985). Several of these studies have however included patients receiving adjuvant therapy.

The absence of tumour ER in patients with advanced disease is an acknowledged predictor of poor response to endocrine therapy (McGuire *et al.*, 1975; Roberts *et al.*, 1978; Allegra *et al.*, 1980; Cant *et al.*, 1985). It was noted by our group that remission rates improve with increasing concentrations of ER (Campbell *et al.*, 1981*a*). We also found an association between the site of initial metastases and ER content of tumour tissue; ER positive tumours preferentially metastasise to bone (Campbell *et al.*, 1981*b*).

We now report our long term findings from a large series of patients with a minimum follow up of 35 months and a maximum follow up of 145 months (median = 84 months). None of the patients considered for analysis received adjuvant therapy of any kind and in all cases the ER status was recorded in the primary tumour.

### Patients and methods

The oestrogen receptor content of primary tumour tissue was measured from 753 patients receiving treatment for operable breast cancer between January 1974 and March 1983. These constitute all women in whom the tumour ER status is known, from 1,000 consecutively treated patients under the care of one surgeon (RWB).

A series of other factors were simultaneously recorded at mastectomy as part of an ongoing project (Nottingham/Tenovus Institute) to study potential prognostic factors for 'early' breast cancer (Haybittle *et al.*, 1982).

No patients in the Nottingham series received adjuvant hormone therapy although a small number received cytotoxic agents immediately after operation ($n = 49$). These patients have been excluded leaving 704 patients for further analysis.

All patients presented under 70 years of age and received either simple/subcutaneous mastectomy ($n = 570$) or lumpectomy and irradiation ($n = 134$) as primary treatment. In the latter group the field of irradiation was confined to the breast and did not include regional nodes. The menopausal status was recorded at presentation and confirmed using LH and FSH levels in women who had undergone previous hysterectomy or were within 5 years of natural cessation of menstruation.

Disease was staged at mastectomy using a triple node biopsy technique (Blamey *et al.*, 1980). In brief one node was sampled from around the axillary vein (apical), one from the low axilla and one from the internal mammary chain. Patients were staged A: with no nodal involvement, B: with

Correspondence: M.R. Williams.
Received 27 May 1986 and in revised form 25 July 1986.

low axillary involvement and C: if either apical or internal mammary nodes were proved to contain tumour on histological examination.

Tumour size was measured from the fresh mastectomy specimen and a sample of the tumour was 'flash' frozen in liquid nitrogen prior to storage at −190°C until assay.

Assays for ER were performed using the Dextran coated charcoal method and tumours with an ER content in excess of 5 fmol mg$^{-1}$ cytosolic protein were classified as ER positive (Nicholson *et al.*, 1981).

Histological grade was assessed in all tumours under the supervision of one pathologist (CWE) using a modification of Bloom and Richardson's criteria (Elston *et al.*, 1980).

Patients were followed in a post mastectomy clinic at three monthly intervals for 18 months, six monthly intervals for three years and annually thereafter.

The disease free interval was recorded as the time to presentation of local or regional recurrence requiring treatment (DXT or excision of axillary nodes) or, in their absence, to the development of distant metastases.

The relationship between ER status and the sites of first presentation of metastases was examined in 290 patients developing distant metastases after grouping into three categories: A: bone metastases, B: lung metastases and C: visceral metastases (liver, brain, intra abdominal). In all these patients systemic therapy had not been prescribed for recurrent local or regional disease before the symptomatic presentation of distant metastases.

Survival after the initiation of therapy was examined in all patients ($n = 204$) receiving endocrine therapy as initial treatment for distant metastases after dividing patients according to the ER status and histological grade of primary tumour tissue. Again all patients receiving prior systemic therapy were excluded.

Two hundred and thirty-five patients with assessable disease received endocrine therapy as initial treatment for local or systemic recurrence. One hundred and eighty-eight of these received endocrine treatment for distant metastases and the remainder for progression of locoregional disease despite radiotherapy.

Endocrine therapy consisted of oophorectomy in premenopausal and Tamoxifen in post-menopausal patients. Criteria for response were strict and followed UICC and BBG guidelines, requiring a minimum remission of six months duration (British Breast Group, 1974; Hayward *et al.*, 1977). External review of response was obtained (Dr A. Howell, Christie Hospital, Manchester).

Analysis of the survival times and DFIs of the patients was performed by life table analysis and the significance of differences between selected groups was assessed using log rank analysis (Peto *et al.*, 1977).

### Results

The overall incidence of ER positivity in this series was 57%.

### *1. Associations between ER status and histological grade, lymph node stage, menopausal status and tumour size*

No correlation was found between ER status and lymph node stage or tumour size. A highly significant relationship existed between ER status and histological grade, ER activity occurred more frequently in well differentiated tumours (Table I). There was also a significant relationship between ER and menopausal status, ER positive tumours occurring more frequently in patients presenting after the menopause. Fifty-one percent of premenopausal patients presented with ER positive tumours compared with 59% of those post-menopausal at presentation ($\chi^2 = 4.77$; $P < 0.05$).

**Table I** ER status *versus* histological grade

| | *Grade* | | |
|---|---|---|---|
| | *I* | *II* | *III* |
| ER positive | 87 | 168 | 146 |
| ER negative | 31 | 92 | 180 |

$\chi^2 = 39.5$; $P < 0.001$.

### *2. ER status, DFI and survival*

When all patients were considered no overall association was found between ER status and DFI ($\chi^2 = 0.97$; 1 df; $P > 0.1$). However in patients with positive lymph nodes at mastectomy a significantly prolonged DFI occurred in those with ER positive tumours (Figure 1). When only patients developing a recurrence within 36 months from mastectomy were examined a significantly prolonged DFI again occurred in those with ER positive tumours (not illustrated, $P < 0.05$).

A weak correlation between patients with ER positive tumours and prolonged survival was maintained throughout the entire study period (Figure 2). This became more marked when patients with positive nodes were examined separately and was maintained after further subdivision according to histological grade (Figure 3). ER positivity conferred no significant survival advantage in lymph node negative patients (Figure 4). The overall survival advantage for all ER positive patients was lost after subdivision according to histological grade (ER positive *versus* ER negative: grade I & II: $\chi^2 = 0.68$; 1 df; $P > 0.5$; grade III: $\chi^2 = 0.29$; 1 df; $P > 0.05$).

| | | | | | | | | | | | | | | | | |
|---|---|---|---|---|---|---|---|---|---|---|---|---|---|---|---|---|
| A | 185 | 176 | 162 | 156 | 148 | 137 | 118 | 103 | 86 | 75 | 63 | 62 | 52 | 50 | 42 | 38 |
| B | 216 | 211 | 200 | 190 | 174 | 158 | 140 | 122 | 110 | 96 | 74 | 69 | 52 | 48 | 35 | 33 |
| C | 185 | 170 | 148 | 128 | 112 | 104 | 95 | 84 | 68 | 58 | 44 | 40 | 29 | 25 | 14 | 12 |
| D | 118 | 103 | 79 | 58 | 45 | 39 | 30 | 24 | 19 | 14 | 9 | 8 | 6 | 6 | 3 | 3 |

**Figure 1** Disease free interval ER positive *versus* ER negative tumours after division according to lymph node status.

**Figure 2** Overall survival ER positive *versus* ER negative tumours.

**Figure 3** Overall survival in lymph node positive patients after division according to histological grade and ER status.

**Figure 4** Overall survival ER positive *versus* ER negative tumours after division according to lymph node status.

*3. ER status and value, response to endocrine therapy and site of initial distant metastasis.*

A strong correlation was found between response to endocrine therapy and ER status. The overall response rate in this series was 23%. Thirty-two percent of patients with ER positive tumours subsequently responded while only 10% of those with ER negative tumours remained in remission 6 months after commencing treatment (Table II). Response rates in ER positive patients improved with increasing ER concentrations, ranging between 19% and 61% in patients with a tumour ER concentration below 40 and above 200 fmol mg$^{-1}$ cytosolic protein respectively (Figure 5).

**Table II** ER and histological grade *versus* endocrine response

| | *Response* | *No response* | *Response rate* |
|---|---|---|---|
| ER positive | 44 | 94 | 32% |
| ER negative | 10 | 87 | 10% |
| Grades I & II | 37 | 56 | 40% |
| Grade III | 17 | 125 | 12% |

ER *versus* response $\chi^2 = 15$; $P < 0.001$. Grade *versus* response $\chi^2 = 24.6$; $P < 0.001$.

**Figure 5** Response as a function of ER concentration.

The site of first documented distant recurrence also correlated well with ER status. One hundred and sixty-five patients with ER positive tumours developed distant metastases before receiving systemic therapy of any kind. One hundred and eighteen (72%) of these had bone involvement at diagnosis while 38 (23%) had visceral involvement. Of 125 patients developing metastases from ER negative tumours, 56 (45%) had visceral involvement and 57 (46%) presented with bone metastases (Table III).

Histological grade of primary tumour tissue was also significantly related to site of initial metastasis and subsequent response to endocrine therapy (Tables II & III).

By combining ER status and histological grade, a group of patients (grade I and II, ER positive: 28% of total) were identified with a response rate of 46%. In contrast only 14%

**Table III** ER and histological grade *versus* initial site of metastases

| | *Site of metastases* | | | *Total patients* |
|---|---|---|---|---|
| | *Bone* | *Lung* | *Visceral* | |
| ER positive | 118 (72%) | 64 (39%) | 38 (23%) | 165 |
| ER negative | 57 (46%) | 39 (31%) | 56 (45%) | 125 |
| Grades I & II | 88 (72%) | 37 (30%) | 25 (20%) | 122 |
| Grade III | 87 (52%) | 66 (39%) | 94 (56%) | 168 |

Percentage of 'total patients' with involvement at each site shown in brackets. Numbers at each site do not summate to 'total patients' due to metastatic involvement in more than one site in many patients.

of all other patients responded and in the 29% of assessable patients with grade III, ER negative tumours, less than 5% remained in remission after 6 months therapy.

*4. ER status and survival after endocrine therapy for distant metastasis*

A clear relationship existed between patients with ER positive primary tumours and prolonged survival after the onset of distant metastasis. Of 122 patients with ER positive tumours receiving first line endocrine therapy 50% were alive at 18 months compared with 18% of 82 patients with ER negative tumours receiving similar treatment (Figure 6).

When survival after symptomatic presentation of metastases was examined in patients showing objective signs of response, those with ER positive tumours had a clear survival advantage over the few patients with ER negative tumours. This did not occur in patients failing to respond to endocrine therapy where there was little survival advantage

**Figure 6** Survival *after metastases*: ER positive *versus* ER negative tumours.

| | | | | | | | | | | | | |
|---|---|---|---|---|---|---|---|---|---|---|---|---|
| A. | 32 | 32 | 27 | 21 | 12 | 10 | 8 | 7 | 6 | 3 | 3 | 2 |
| B. | 9 | 9 | 6 | 3 | 3 | 2 | 1 | 1 | 1 | 1 | 1 | 0 |
| C. | 80 | 53 | 31 | 19 | 12 | 5 | 3 | 2 | 1 | 1 | 1 | 0 |
| D. | 67 | 28 | 18 | 7 | 6 | 3 | 2 | 2 | 0 | 0 | 0 | 0 |

**Figure 7** Survival *after metastases*: ER positive *versus* ER negative tumours in patients showing response or no response to endocrine therapy.

for patients with ER positive tumours (Figure 7). Interestingly when patients were grouped according to histological grade and ER status, the major survival advantage after metastases was confined to patients with well differentiated ER positive tumours (grade I and II). Patients with poorly differentiated (grade III) ER positive tumours had similar survival characteristics to all those with ER negative tumours (Figure 8).

### Discussion

This study confirms the importance of ER status when used alone as a prognostic indicator in patients presenting with lymph node positive primary breast cancer. The incidence of ER positivity is similar to that reported by Alanko (63%) and Raemaekers (66%) but is lower than that found in other series: Parl (76%); Aamdal (72%) and Howell (70%). Our exclusion criteria for patients presenting over 70 years of age may in part account for these differences.

The long term discrimination in overall survival achieved by oestrogen receptor status is lost when all patients are grouped according to histological grade. This agrees with our previous finding that the prognostic importance of ER is largely dependent upon its association with grade. ER status does not appear as an independently significant prognostic factor when included with grade in a multivariate analysis (Haybittle *et al.*, 1982). If histological grade is not assessed then the use of ER combined with node stage provides a good prognostic discriminant. The prognostic importance of ER is not however entirely dependent upon its association with grade as in lymph node positive patients, even after further subdivision according to histological grade, survival is significantly prolonged in patients with ER positive tumours. After longer follow-up, with more events, this advantage for patients with ER positive tumours may also appear in patients lymph node negative at mastectomy.

| | | | | | | | | | | | |
|---|---|---|---|---|---|---|---|---|---|---|---|
| A | 58 | 49 | 37 | 30 | 20 | 13 | 8 | 8 | 6 | 4 | 4 |
| B | 63 | 43 | 26 | 15 | 7 | 4 | 3 | 1 | 1 | 1 | 1 |
| C | 28 | 18 | 15 | 5 | 5 | 3 | 3 | 2 | 1 | 1 | 1 |
| D | 55 | 21 | 11 | 6 | 5 | 3 | 1 | 1 | 0 | 0 | 0 |

**Figure 8** Survival *after metastases*: ER positive *versus* ER negative tumours after division according to histological grade.

Our findings are at variance with those reported by Alanko *et al.* (1984) and Raemaekers *et al.* (1985) who found no association between DFI (or survival) and ER status even after stratification according to lymph node stage at mastectomy. The mean follow-up of patients in these series was 41 and 76 months respectively. They suggest that previous reports, showing an advantage for patients with ER positive tumours, may have been influenced by the addition of adjuvant therapy at mastectomy. In contrast Howat *et al.* (1985) reported a significant improvement in relapse free survival for patients with positive axillary nodes when ER was present in primary tumour. However this advantage for ER positive patients was confined to those with relatively few nodes (1 to 3) involved at mastectomy.

The major advantage for patients with ER positive tumours occurs after the onset of recurrent disease, as DFI is unaffected by ER status overall. This suggests a survival advantage conferred on this group by either the effect of treatment response or the site of initial metastasis. Both these factors are seen to correlate with ER status. These findings are in agreement with those reported by Howell *et al.* (1984) and Howat *et al.* (1985) where, despite no overall difference in disease free interval, improved survival after first relapse of disease was noted in patients with ER positive tumours.

In our series in those patients presenting with poorly differentiated (grade III) carcinoma there is little advantage in terms of survival after the appearance of metastases for those with ER positive tumours. This again underlines the importance of histological grade, even in advanced disease. The improved survival after metastases seen only in patients with well differentiated (grade I and II) ER positive tumours may suggest that this group has an advantage in terms of tumour growth rate (as reflected by tumour grade) allowing endocrine therapies to have their maximal effect. In addition the association between tumours of poor histological differentiation and visceral metastases may be an important factor.

The low response rate of 23% reflects our strict assessment criteria. ER negativity is confirmed as a predictor of poor response to subsequent endocrine manipulation. However it is to be noted that 10% of patients still responded to endocrine treatment despite the absence of ER in primary tumour tissue. A strong association is also found between histological grade and therapeutic response, in agreement with reports from another centre (Millis *et al.*, 1981; Masters *et al.*, 1986). This in part may indicate that patients with low grade tumours, which have a naturally slow growth rate, have time to maintain a remission for the required six months defined by the response criteria adopted. It is to be noted that tumour grading has been performed under the supervision of one experienced pathologist (CWE) with a particular interest in this field. In this situation histological differentiation has similar potential to ER status in predicting subsequent response to endocrine therapy using BBG criteria. The combination of ER status and histological grade is able to achieve the greatest discrimination between those patients most likely to benefit from hormone manipulation and those in whom disease progresses.

It is of great interest that, even in the presence of objective signs of response, patients with ER negative tumours have poor survival after symptoms from metastases first occur. This may represent a misclassification of response in these patients as, indeed, their long term survival on therapy was similar to all those progressing despite treatment. This increases the confidence with which ER status predicts a failure of endocrine treatment in terms of survivial after metastases.

Therapeutic implications in the management of patients with advanced breast cancer can be drawn from these findings. By combining the results of ER assays with hisotological grade a group of patients comprising 28% of the total is identified (ER positive, grade I and II). These patients enjoy relatively good survival after commencing first line endocrine therapy which exceeds 40% at three years. In this situation therefore endocrine therapy should be instituted in all cases. However patients with ER positive grade III tumours and those with ER negative tumours have equally poor survival characteristics after metastases are confirmed. In these situations the addition of alternative treatments may be indicated at an early stage. We suggest that where possible an accurate assessment of histological grade should be performed at the same time as oestrogen receptor analyses to identify those patients who behave favourably after the initiation of endocrine treatment.

In conclusion a strong correlation is found between ER status in primary breast carcinoma and histological grade. ER status relates weakly to overall survival but not to the disease free interval. ER status relates strongly to both DFI and overall survival when lymph node positive patients are examined separately. These findings in lymph node positive patients are maintained even after further subdivision according to histological grade. ER status relates to the site of initial metastases and both ER status and quantitative levels relate to subsequent response to endocrine therapy.

M.R. Williams is supported by the Tenovus Institute, Cardiff.

### References

AAMDAL, S., BORMER, O., JORGENSEN, O. & 5 others. (1984). Estrogen receptors and long term prognosis in breast cancer. *Cancer*, **53**, 2525.

ALANKO, A., HEINONEN, E., SCHEININ, T.M., TOLPPANEN, E.-M., VIHKO, R. (1984). Oestrogen and progesterone receptors and disease-free interval in primary breast cancer. *Br. J. Cancer*, **50**, 667.

ALLEGRA, J.C., LIPPMAN, M.E., SIMON, R. & 6 others. (1979). Association between steroid hormone receptor status and disease free interval in breast cancer. *Cancer Treat. Rep.*, **63**, 1271.

ALLEGRA, J.C., LIPPMANN, M.E., THOMPSON, E.B. & 7 others. (1980). Estrogen receptor status: An important variable in predicting response to endocrine therapy in metastatic breast cancer. *Eur. J. Cancer*, **16**, 323.

BISHOP, H.M., BLAMEY, R.W., ELSTON, C.W., HAYBITTLE, J.L., NICHOLSON, R.I. & GRIFFITHS, K. (1979). Relationship of oestrogen-receptor status to survival in breast cancer. *Lancet*, **ii**, 283.

BLAMEY, R.W., BISHOP, H.M., BLAKE, J.R.S. & 5 others. (1980). Relationship between primary breast tumor receptor status and patient survival. *Cancer*, **46**, 2765.

BRITISH BREAST GROUP. (1974). Assessment of response to treatment in advanced breast cancer. *Lancet*, **ii**, 38.

CALDAROLA, L., CALDERINI, P., VOLTERRANI, P., DI CARLO, F. & GAGLIA, P. (1983). The correlation between estrogen receptor status, axillary-node metastases and disease free interval after surgery in primary breast cancer. *Ital. J. Surg. Sci.*, **13**, 179.

CAMPBELL, F.C., BLAMEY, R.W., ELSTON, C.W. & 4 others. (1981*a*). Quantitative oestradiol receptor values in primary breast cancer and response of metastases to endocrine therapy. *Lancet*, **ii**, 1317.

CAMPBELL, F.C., BLAMEY, R.W., ELSTON, C.W., NICHOLSON, R.I., GRIFFITHS, K. & HAYBITTLE, J.L. (1981*b*). Oestrogen-receptor status and sites of metastasis in breast cancer. *Br. J. Cancer*, **44**, 456.

CANT, E.M., HORSFALL, D. & KEIGHTLEY, D.D. (1985). Value of hormone receptors in the management of breast cancer – 1. Advanced breast cancer. *Aust. N.Z. J. Surg.*, **55**, 121.

COOKE, T., GEORGE, D., SHIELDS, R., MAYNARD, P. & GRIFFITHS, K. (1979). Oestrogen receptors and prognosis in early breast cancer. *Lancet*, **ii**, 995.

ELSTON, C.W., BLAMEY, R.W., JOHNSON, J., BISHOP, H.M., HAYBITTLE, J.L. & GRIFFITHS, K. (1980). The relationship of oestradiol receptor (ER) and histological tumour differentiation with prognosis in human primary breast carcinoma. In *Breast Cancer – Experimental and Clinical Aspects*, Mouridsen & Palshof (eds) p. 59. Pergamon Press: Oxford.

HAYBITTLE, J.L., BLAMEY, R.W., ELSTON, C.W. & 5 others. (1982). A prognostic index in primary breast cancer. *Br. J. Cancer*, **45**, 361.

HAYWARD, J.L., CARBONE, P.P., HEUSON, J.-C., KUMAOKA, S., SEGALOFF, A. & RUBENS, R.D. (1977). Assessment of response to therapy in advanced breast cancer. *Cancer*, **39**, 1289.

HILF, R., FELDSTEIN, M.L., GIBSON, S.L. & SAVLOV, E.D. (1980). The relative importance of estrogen receptor analysis as a prognostic factor for recurrence or response to chemotherapy in women with breast cancer. *Cancer*, **45**, 1993.

HOWAT, J.M.T., HARRIS, M., SWINDELL, R. & BARNES, D.M. (1985). The effect of oestrogen and progesterone receptors on recurrence and survival in patients with carcinoma of the breast. *Br. J. Cancer*, **51**, 263.

HOWELL, A., BARNES, D.M., HARLAND, R.N.L. & 6 others. (1984). Steroid-hormone receptors and survival after first relapse in breast cancer. *Lancet*, **i**, 588.

KINNE, D.W., ASHIKARI, R., BUTLER, A., MENENDEZ-BOTET, C., ROSEN, P.P. & SCHWARTZ, M. (1981). Estrogen receptor protein in breast cancer as a predictor of recurrence. *Cancer*, **47**, 2364.

KNIGHT, W.A., LIVINGSTON, R.B., GREGORY, E.J. & McGUIRE, W.L. (1977). Estrogen receptor as an independent prognostic factor for early recurrence in breast cancer. *Cancer Res.*, **37**, 4669.

MASTERS, J.R.W., MILLIS, R.R. & RUBENS, R.D. (1986). Response to endocrine therapy and breast cancer differentiation. *Breast Cancer Res. Treat.*, **7**, 31.

MAYNARD, P.V., BLAMEY, R.W., ELSTON, C.W., HAYBITTLE, J.L. & GRIFFITHS, K. (1978*a*). Estrogen receptor assays in primary breast cancer and early recurrence of the disease. *Cancer Res.*, **38**, 4292.

MAYNARD, P.V., DAVIES, C.J., BLAMEY, R.W., ELSTON, C.W., JOHNSON, J. & GRIFFITHS, K. (1978*b*). Relationship between oestrogen-receptor content and histological grade in human primary breast tumours. *Br. J. Cancer*, **38**, 745.

McGUIRE, W.L., CARBONE, P.P., SEARS, H.E. & ESCHER, G.C. (1975). In *Estrogen Receptors in Human Breast Cancer*, McGuire *et al.* (eds) p. 1. Raven Press: New York.

MILLIS, R.R., RUBENS, R.D., MASTERS, J.R.W. & MINTON, M.J. (1981). Histological grade and response to endocrine therapy in breast cancer. *Lancet*, **i**, 101.

NEIFIELD, J.P. LAWRENCE, W. Jr., BROWN, P.W., BANKS, W.L. & TERZ, J.J. (1982). Estrogen receptors in primary breast cancer. *Arch. Surg.* **117**, 753.

NICHOLSON, R.I., CAMPBELL, F.C., BLAMEY, R.W., ELSTON, C.W., GEORGE, D. & GRIFFITHS, K. (1981). Steroid receptors in early breast cancer: Value in prognosis. *J. Steroid Biochem.*, **15**, 193.

OSBORNE, C.K., YOCHMOWITZ, M.G., KNIGHT, W.A. III & McGUIRE, W.L. (1980). The value of estrogen and progesterone receptors in the treatment of breast cancer. *Cancer*, **46**, 2884.

PARL, F.F., SCHMIDT, B.P., DUPONT, W.D. & WAGNER, R.K. (1984). Prognostic significance of estrogen receptor status in breast cancer in relation to tumour stage, axillary node metastases, and histological grading. *Cancer*, **54**, 2237.

PATERSON, A.H., ZUCK, P.V., SZAFRAN, O., LEES, A.W. & HANSON, J. (1982). Influence and significance of certain prognostic factors on survival in breast cancer. *Eur. J. Cancer Clin. Oncol.*, **18**, 937.

PETO, R., PIKE, M.C., ARMITAGE, P. & 7 others. (1977). Design and analysis of randomised clinical trials requiring prolonged observation of each patient. II Analysis and examples. *Br. J. Cancer*, **35**, 1.

RAEMAEKERS, J.M.M., BEEX, L.V.A.M., KOENDERS, A.J.M. & 5 others. (1985). Disease-free interval and estrogen receptor activity in tumor tissue of patients with primary breast cancer: Analysis after long-term follow up. *Breast Cancer Res. Treat.*, **6**, 123.

ROBERTS, M.M., RUBENS, R.D., KING, R.J.B. & 4 others. (1978). Oestrogen receptors and the response to endocrine therapy in advanced breast cancer. *Br. J. Cancer*, **38**, 431.

SAMAAN, N.A., BUZDAR, A.U., ALDINGER, K.A. & 4 others. (1981). Estrogen receptor: A prognostic factor in breast cancer. *Cancer*, **47**, 554.